# LncRNA expression profile and ceRNA analysis in tomato during flowering

**DOI:** 10.1371/journal.pone.0210650

**Published:** 2019-01-17

**Authors:** Zhenchao Yang, Chengcheng Yang, Zhengyan Wang, Zhao Yang, Danyan Chen, Yongjun Wu

**Affiliations:** College of Horticulture, College of Life Sciences, Northwest A&F University, Yangling, Shaan Xi, China; Harvard Medical School, UNITED STATES

## Abstract

Long non-coding RNAs (lncRNAs) are a class of non-coding RNAs that play essential regulatory roles in various developmental processes and stress responses. However, the functions of lncRNAs during the flowering period of tomato are largely unknown. To explore the lncRNA profiles and functions during flowering in tomato, we performed strand-specific paired-end RNA sequencing of tomato leaves, flowers and roots, with three biological replicates. We identified 10919 lncRNAs including 248 novel lncRNAs, of which 65 novel lncRNAs were significantly differentially expressed (DE) in the flowers, leaves, and roots. The Gene Ontology (GO) and Kyoto Encyclopedia of Genes and Genomes (KEGG) analyses were carried out to identify the cis target gene of DE lncRNAs. The results showed that the lncRNAs might play an important role in the growth, development, and apoptosis of flowering tomato plant by regulating the formation of intima in flower tissues, binding to various molecules, influencing metabolic pathways, and inducing apoptosis. Moreover, we identified the interaction between 32, 78, and 397 kinds of miRNAs, lncRNAs, and mRNAs. The results suggest that the lncRNAs can regulate the expression of mRNA during flowering period in tomato by forming competitive endogenous RNA, and further regulate various biological metabolism pathways in tomato.

## Introduction

Long noncoding RNA (lncRNA) is a class of noncoding RNA, which are approximately 200 nt to 100 kb long [1]. Pachnis et al. [2] discovered the first lncRNA H19 associated with X chromosome, through cloning, sequencing, and gene mapping. Initially, lncRNA was assumed to be a kind of transcriptional noise like other noncoding RNAs. Further research in recent years showed that lncRNA has important roles in biological processes [3]. Studies on human or animal lncRNAs are more extensive than on plant lncRNAs [4–5]. Studies have showed that the lncRNAs can regulate the expression of cis-acting or trans-acting genes, and the regulation of expression level of cis-acting genes might represent a widespread function of lncRNAs [3]. The lncRNA upstream of a gene might interact with the promoter or other cis-acting elements of co-expressing genes to regulate gene expression at the transcriptional or post-transcriptional level [6–7]. The lncRNA overlap with transcription factor binding sites; thus, the transcription of lncRNA can prevent transcription factors from binding to this site [3, 6–7]. Moreover, lncRNA near the binding site of a transcription factor could enhance the binding of the transcription factor to the site [7]. In general, lncRNA can be classified as sense, natural antisense, bidirectional, intronic, and intergenic lncRNAs [2–3]. Different types of lncRNAs have different regulatory roles. Plant lncRNAs participate in processes such as growth, development, and stress response. For example, overexpression of lncRNA npc48 in Arabidopsis increased the rosette diameter and leaf serration, and delayed the flowering time compared with those in wild-type plants [8]. In total, 664 lncRNAs were identified in response to drought stress in maize leaves [9]; among them, 567 lncRNAs were up-regulated, whereas 97 lncRNAs were down-regulated. More lncRNAs have been reported in different plants through deep sequencing, such as those in *Arabidopsis* [10–18], *Oryza* [19–22], Zea mays L. [23–24], *Medicago sativa* L. [25], *Brassica* [26], *Populus trichocarpa* [27], *Triticum aestivum L*. [28], and *Malus* [29].

Tomato (*Solanum lycopersicum*), an important vegetable crop, is rich in proteins, lipids, carbohydrates, vitamin A, vitamin B1, vitamin B2, vitamin C, calcium, iron, phosphorus, and other minerals. Some tomato lncRNAs have been reported recently. Wang et al. [30] identified lncRNAs in tomato plants infected with tomato yellow leaf curl virus (TYLCV), and found that the expression of slylnc0049 and slylnc0761 could inhibit the infection of TYLCV in tomato. Li et al. [31] reported that low expression of lncRNA 1459 could inhibit fruit ripening in tomato and that altered expression of other lncRNAs affected fruit ripening. Flowering period is an important stage of plant development, which influences fruit formation and production; lncRNA can regulate flowering. COOLAIR and COLDAIR are two kinds of lncRNA, and they inhibit the expression of *FLC* in the lncR2Epi regulation pathway under cold stress [32–33]. Further, COLDAIR can affect flowering by inhibiting *FLOWERING LOCUS C (FLC)* expression during vernalization [4, 34–35]. *FLC* is a kind of MADS box transcriptional factor, which inhibits flowering in cold environments [36]. COOLAIR affects the decrease in H3K36me3 or H3K4me2 at *FLC* [34, 37]; however, COLDAIR and PRC2 together promote the accumulation of H3K27me3 at *FLC* [35]. Therefore, studies on tomato flowering-related lncRNAs are of significance to further understand the regulation mechanism of lncRNA involved in flowering tomato development.

Recent studies have showed that lncRNA can act as endogenous target mimics (eTMs) and involve several kinds of physiological activities similar to that of competitive endogenous RNA (ceRNA). Deng et al. [38] studied the transcriptome of *Gossypium hirsutum* under NaCl stress and identified lnc_973 and lnc_253 as ceRNAs that regulate the expression of ghr-miR399 and ghr-156e in response to NaCl stress. Xu et al. [39] identified lncRNAs from phosphorus-deficient rice and constructed a ceRNA regulatory network of rice root under phosphorus deficiency. Recently, lncRNA function was studied by constructing the lncRNA-miRNA-mRNA regulatory network. He et al. [40] discussed lncRNA function through the lncRNA-miRNA-mRNA axes in vascular pathophysiology and cardioprotection. Zhu et al. [41] analyzed the ceRNA network of 17 lncRNAs, 840 mRNAs, and genomic miRNAs of maize. They discovered seven novel lncRNAs, which might act as a ceRNA, and elucidated that some ceRNAs together affected maize seed development and metabolic processes. Overall, lncRNA might participate in various biological processes, by acting as a ceRNA.

In the present study, we analyzed the lncRNA expression profile of the leaves, roots, and flowers of tomato by deep sequencing; identified new and differentially expressed (DE) lncRNAs; analyzed lncRNA expression by quantitative reverse transcription-polymerase chain reaction (qRT-PCR); predicted potential function of lncRNAs and their cis-acting targets; and acquired the lncRNA-related pathway and gene ontology information. We also constructed a ceRNA network based on the interaction between lncRNA, miRNA, and mRNA.

## Materials and methods

### Samples collection

Tomato (*S*. *lycopersicum* Mill. ‘Ailsa Craig’) was used in this study. All the plants were grown in a greenhouse under 16 h-day/8 h-night cycles, 26°C/18 °C day/night temperature, 80% humidity, and 250 μmol m^-2^ s^-1^ light intensity. When tomato plants flowered for the first time, the leaves (third leaf from the top), flowers (without sepals), and roots (mixed sample of all root tissues) were collected as experimental materials. After sampling, all the tissues were immediately frozen using liquid nitrogen and stored at -80 °C.

### LncRNA library construction and RNA sequencing

RNA was extracted from the leaves, flowers, and roots from three flowering tomato plants (there were three biological replicates per tissue). The total RNA from all the samples was extracted using TRIzol (Invitrogen, Carlsbad, CA, USA). RNAs of satisfactory quality were treated using the Ribo-Zero Gold kit (Epicentre, San Diego, CA, USA) to eliminate rRNAs. NEBNext Ultra Directional RNA (NEB, Beijing, China) was used to construct libraries. Sequencing was performed using Illumina HiSeq 2000 platform, and 150-bp paired-end reads were obtained. mRNA and non-coding RNA were randomly broken into short fragments of length 200–500 bp. The short fragments were used as templates to synthesize the first strand cDNA with six-base random primers and dUTP was used instead of dTTP to synthesize the second strand cDNA. After purification of the synthesized double-stranded cDNA, the terminal repair and A-tail addition were performed, and the sequencing linker was ligated. Furthermore, uracil-N-glycosylase was added to degrade the second strand. The fragment size was selected by agarose gel electrophoresis followed by PCR amplification. The final sequenced library was sequenced using the Illumina sequencing platform.

### Classification of lncRNAs

Based on the positional relationship of the lncRNA transcript to its neighboring gene (RNA partner) transcripts on the chromosome (S1 Fig), we divided the lncRNA into the following two types: genic (the lncRNA transcript overlaps with RNA partner transcript) and intergenic (the lncRNA transcript is located in the intergenic region). Based on the degree of overlap between the lncRNA transcript and the RNA partner transcript and the direction of transcription, the genic lncRNA was further subdivided into overlapping (the lncRNA transcript partially overlaps with the RNA partner transcript), containing (the RNA partner transcripts are contained in the lncRNA transcripts), and nested (the lncRNA transcripts are contained in the RNA partner transcripts); and the intergenic lncRNA was further subdivided into same strand (the lncRNA and RNA partner are transcribed from the same strand and have the same transcriptional direction), convergent (the lncRNA and RNA partner are transcribed from the positive and negative strands, respectively, and the direction is convergent), and divergent (the lncRNA and RNA partner are transcribed from the positive and negative strands, respectively, and the transcriptional direction is divergent).

### Transcriptome assembly and novel lncRNA prediction

Based on the results of sequence reads and alignments with the reference genome (https://www.ncbi.nlm.nih.gov/genome/?term=tomato), we re-constructed transcripts using String Tie [42].The re-constructed transcripts from nine samples were compared mutually using cuffcompare [43] and the redundant transcripts were removed. Thereafter, filtering and screening of the re-constructed transcripts were performed according to the method of Prensner et al. [44]. The steps were as follows:

Step 0: Re-constructed transcripts of multiple samples were compared using cuffcompare.
Step 1: Transcripts shorter than 200 bp were filtered.
Step 2: The background transcripts were filtered (the read coverage of each transcript was calculated using cufflinks and the transcripts with read coverage of ≥ 3 were selected).
Step 3: The overlapping transcripts of known lncRNAs and precursor transcripts of mRNAs were filtered (the non-lncRNA genes by comparison with known non-lncRNA genes were filtered out and the transcripts with > 90% similarity to known lncRNAs based on the blast results were removed).
Step 4: The filtered transcripts were predicted using coding potential calculator (CPC) and Pfam. Transcripts with CPC_threshold < 0 and not aligned with Pfam are defined as lncRNA.

We used CPC [45] to analyze the coding ability and Pfam [46] in order to analyze the protein domains; the intersection of multiple steps was chosen as the novel lncRNA data set. The predicted lncRNAs, known lncRNAs, and known genes were combined as a reference sequence set for follow-up analysis.

### Detection of lncRNA by qRT-PCR

The total RNA from each tissue, namely, the leaves, flowers, and roots, of three flowering tomato plants was extracted using TRIzol (Invitrogen) and purified using the RNA purification kit (Promega, Madison, WI USA). The concentration and purity of RNA were measured using Nanodrop (Thermo, Madison, WI, USA), and RNA integrity was detected by agarose gel electrophoresis. DNase was used to eliminate genomic DNA from the total RNA. Two micrograms of total RNA were used for cDNA synthesis using the GOSCRIPT Reverse transcriptional system (Promega). To determine the expression of lncRNA and transcripts of the coding gene, specific primers and SYBR Green PCR Master Mix (Promega) were used to perform qRT-PCR using Bio-Rad CFX manager 3.1 (Bio-Rad, Hercules, CA USA) [47]; actin was used as the standard. The data were analyzed by the 2^-ΔΔCt^ method [48]. All the results are expressed as mean ± standard deviation (SD) of three biological replicates. The primers used for qRT-PCR are listed in S1 Table.

### Gene expression quantification and differential expression analysis

The genomic reads were mapped to the exon regions of the gene using HTseq [49], and then the number of reads per gene was calculated to estimate the gene expression levels. The reads were counted using the reads per kilo bases per million reads (RPKM) formula:

$$RPKM(A)\;=\;\frac{10^{6}C}{NL/10^{3}}$$

where, RPKM(A) represents the expression level of gene A, *C* represents the number of reads aligned only to gene A, *N* is the total number of reads aligned to the reference gene, and *L* is the number of bases in the coding region of gene A.

Differences in lncRNA sequences were analyzed using DEGseq [50] and DESeq2 [51]. Different lncRNAs were screened if the difference multiplier was greater than or equal to 2 and Q-value (or FDR) was less than or equal to 0.01. Hierarchical clustering analysis was carried out using pheatmap package of R 3.5.0. Different color regions represent different clustering group information, and the gene expression patterns in the same group were similar, and the genes might have similar functions or participate in the same biological process.

### Prediction of cis-acting target gene

The expression value (RPKM) of each lncRNA and mRNA was calculated in various tissues. These expression data were used to calculate Pearson correlation coefficient following the method of coding-non-coding gene co-expression (CNC) [52]. We screened for DE lncRNAs and DE mRNAs with high correlation (P < 0.01), and then screened for mRNAs that overlapped with lncRNAs at the genomic position or located 100-kb upstream and downstream of an lncRNA as a candidate target gene for lncRNA regulation. By combining the correlation between lncRNAs and genes, we chose lncRNAs that overlapped at the genomic location or the upstream and downstream 100-kb genes as candidate targets for lncRNA regulation.

### Gene ontology (GO) and Kyoto Encyclopedia of Genes and Genomes (KEGG) enrichment analyses

To account for the effect of selection bias, GOseq [53] was used for the GO enrichment analysis of DE or target genes of DE lncRNAs. The following formula was used to detect statistical enrichment of DE or target genes of DE lncRNAs in the KEGG pathway:

$$P\;=\;1-\sum_{i\;=\;0}^{m-1}\frac{\left(\frac{M}{i})(\frac{N-M}{n-i}\right)}{\left(\frac{N}{n}\right)}$$

where, *N* is the annotation of all genes, *n* is the number of DE genes among *N*, *M* is the number of genes annotated to a specific pathway, *m* is the number of DE genes annotated to a specific pathway *M*.

### CeRNA network analysis

The miRanda and TargetScan [54] assessments were used to identify ceRNAs containing the microRNA response element (MRE). The psRNATarget [55] and psRobot [56] were used to predict whether an lncRNA could be a miRNA target. Among the genes with significant differential expression, ceRNAs with common miRNA binding site were selected to predict the global interaction between miRNA and ceRNA. We removed the false positive ceRNA by verifying the opposite trend of expression between the miRNA and the gene. The co-regulated interaction of miRNA and ceRNA was predicted based on the differential expression of lncRNA in each tissue with the same expression trend as mRNA and the opposite expression trend to miRNA. Cytoscape 3.4.0 was used analyze and visualize the ceRNA network.

## Results

### Genome-wide identification of lncRNAs in tomato during flowering

To comprehensively explore lncRNAs in tomato during flowering, RNA from the leaves, flowers, and roots of flowering tomato plants was extracted for whole-transcriptome strand-specific RNA sequencing (three biological duplicates were performed per organ; SRA accession: PRJNA507058, https://www.ncbi.nlm.nih.gov/sra/PRJNA507058). A total of 81,300,887 raw reads were obtained by sequencing the sample, and 99.98% of these were clean reads (S2 Table). The results showed that both RNA-seq reads and the tomato reference genome were of high quality. We found that an average of 82.76% of the reads could be mapped to the unique location of the genome. Based on the comparison of reads of the genomes studied, the transcripts were reconfigured and assembled using StringTie. Subsequently, nine cDNA libraries of three tomato tissues were established. A total of 54,123 lncRNAs were obtained from nine libraries for subsequent analysis. After filtering out the known lncRNAs, 523 novel lncRNAs were identified for subsequent analysis using CPC and Pfam (S3 Table). These novel lncRNAs might be valuable in the flowering process of tomato.

### Characteristics of tomato lncRNAs

**Fig 1:**
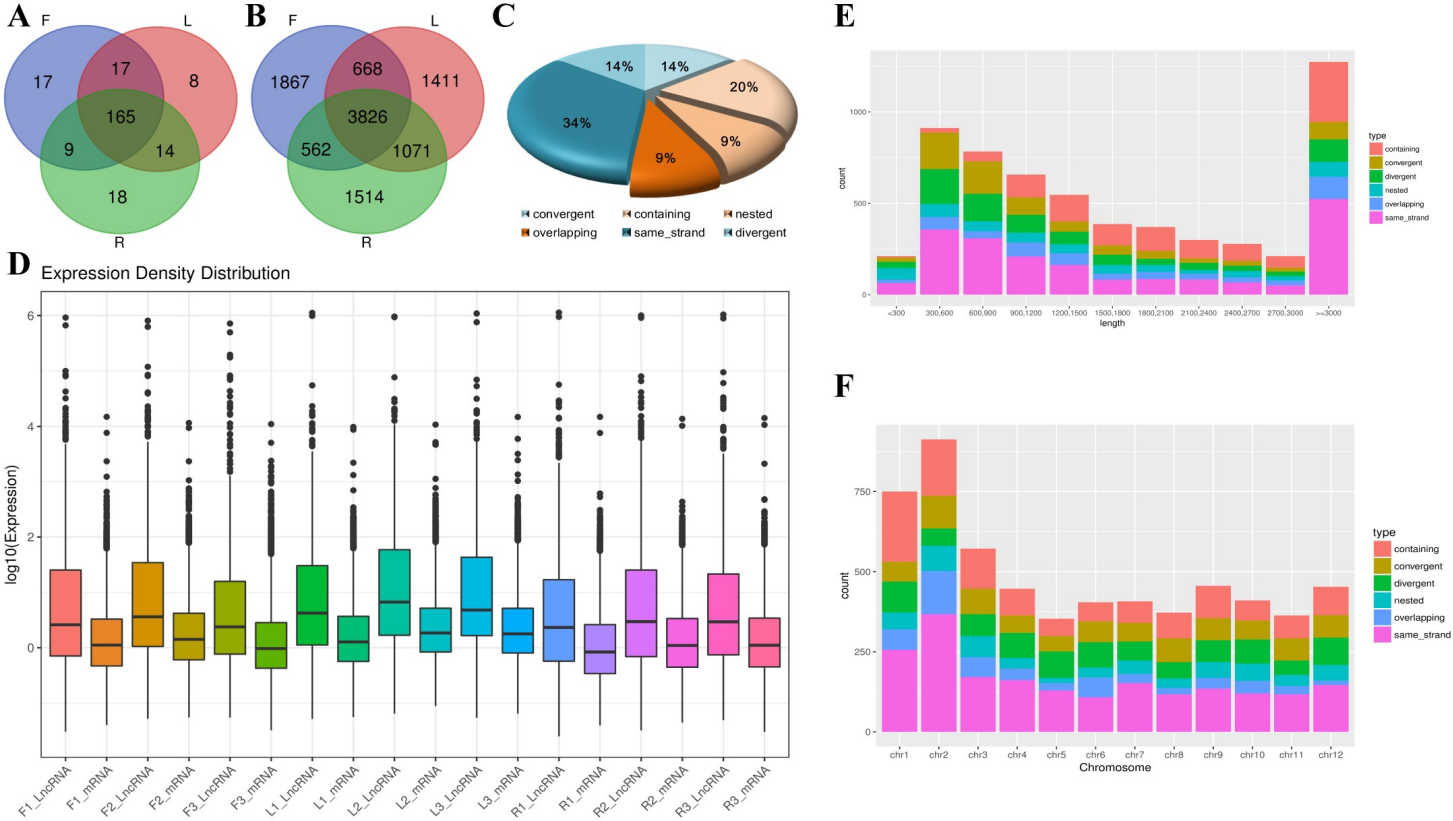
Analysis of tomato lncRNAs. Venn diagram showing the number of all **(A)** and novel **(B)** lncRNAs expressed tissue-specifically in tomato. **(C)**The pie chart shows the tissue-specific distribution of different types of lncRNAs in tomato during flowering; blue indicates intergenic and gold indicates genic lncRNAs. **(D)** The box plots showing the expression level of lncRNA-mRNA pairs. **(E)** Length distribution of tomato lncRNAs. **(F)** The density of lncRNAs in different tomato chromosomes. lncRNAs **(E, F)** are of six types—containing (red), convergent (brown), divergent (green), nested (sky-blue), same_strand (blue), and overlapping (purple).

We performed an expression profiling analysis for the lncRNAs and mRNAs mentioned above. The expression boxplot (Fig 1D) showed that the overall distribution of tomato lncRNA and mRNA expression was consistent and that there were no systematic deviation data. After removing the low-expression lncRNAs (RPKM < 1), 10,919 lncRNAs were discovered from the three tomato tissues studied (S4 Table). Among them, 248 lncRNAs were novel (S5 Table). Thereafter, an intersection analysis of lncRNAs from the three tomato tissues was performed. The results showed that 165 lncRNAs (Fig 1B) among the 3826 lncRNAs (Fig 1A) detected from the three tissues were novel. Moreover, there were 17, 8, and 18 tissue-specific novel lncRNAs in the flowers, leaves, and roots, respectively.

**Fig 2:**
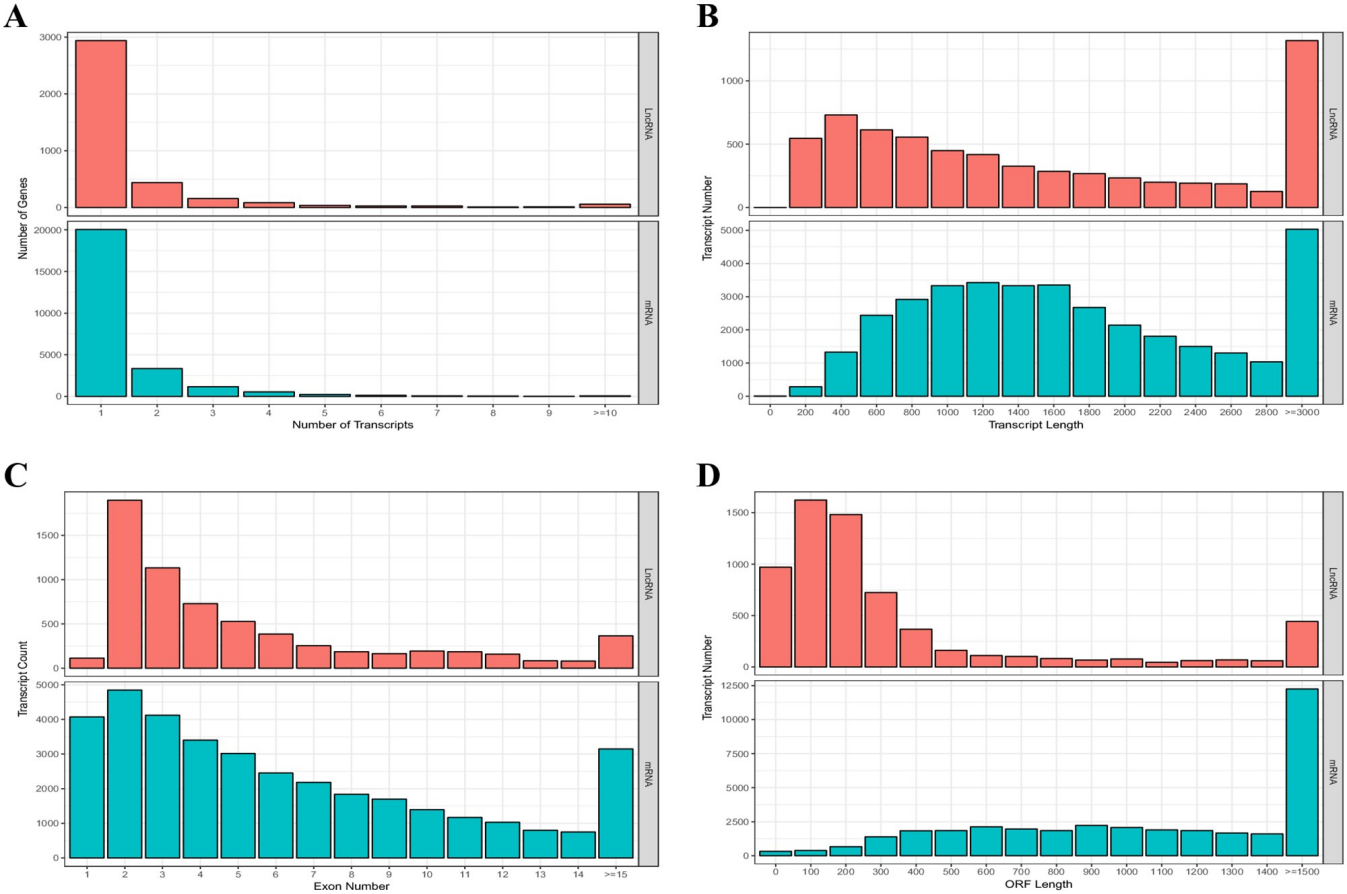
Analysis of transcripts, exons, and ORFs in tomato. **(A)** Number of transcripts, **(B)** length of transcripts, **(C)** number of exons, and **(D)** length of ORFs. The red bar represents the number of lncRNA and the blue bar represents the number of mRNAs.

According to the location of lncRNAs and their adjacent genes, we divided tomato lncRNAs into two types—genic and intergenic. They were further divided into six subclasses (S1 Fig). Among them, the genic lncRNAs can be divided into containing, convergent, and nested types, and the intergenic lncRNAs could be divided into divergent, same_strand, and overlapping types. Compared with that of the genic lncRNAs, the intergenic lncRNAs accounted for a high proportion (62%) of flowering period tomato lncRNAs. The containing type is the main type of genic lncRNAs, and the same-strand type is the main type of intergenic lncRNAs and the most common of the six types (Fig 1C). Moreover, with increase in the length of lncRNAs, the number of lncRNAs decreased (Fig 1D). The same strand-type lncRNAs occupied a higher proportion among the short lncRNAs (< 1500 bp), whereas the containing-type lncRNAs occupied a higher proportion among the longer lncRNAs (Fig 1D). In addition, compared with that of the other chromosomes, chr1 and chr2 were more likely to produce lncRNAs (Fig 1E). A comparison of genomic characteristics of the identified lncRNAs and tomato protein coding genes showed that the total number of transcripts of lncRNAs was less than that of mRNAs (Fig 2A). However, most lncRNAs and mRNAs had only one transcript. Moreover, the lncRNAs were significantly shorter than the mRNAs (Fig 2B). The number of transcripts of lncRNAs with a single exon was relatively less; > 90% of lncRNAs had > 2 exons (Fig 2C). In addition, a significant difference between mRNAs and lncRNAs was the length of the open reading frame (ORF), ranging between 0 and 300 bp. Moreover, > 900 lncRNA transcripts had no ORF (Fig 2D).

### Differences among tissues and verification of lncRNAs from tomato flowers, leaves, and roots

**Fig 3:**
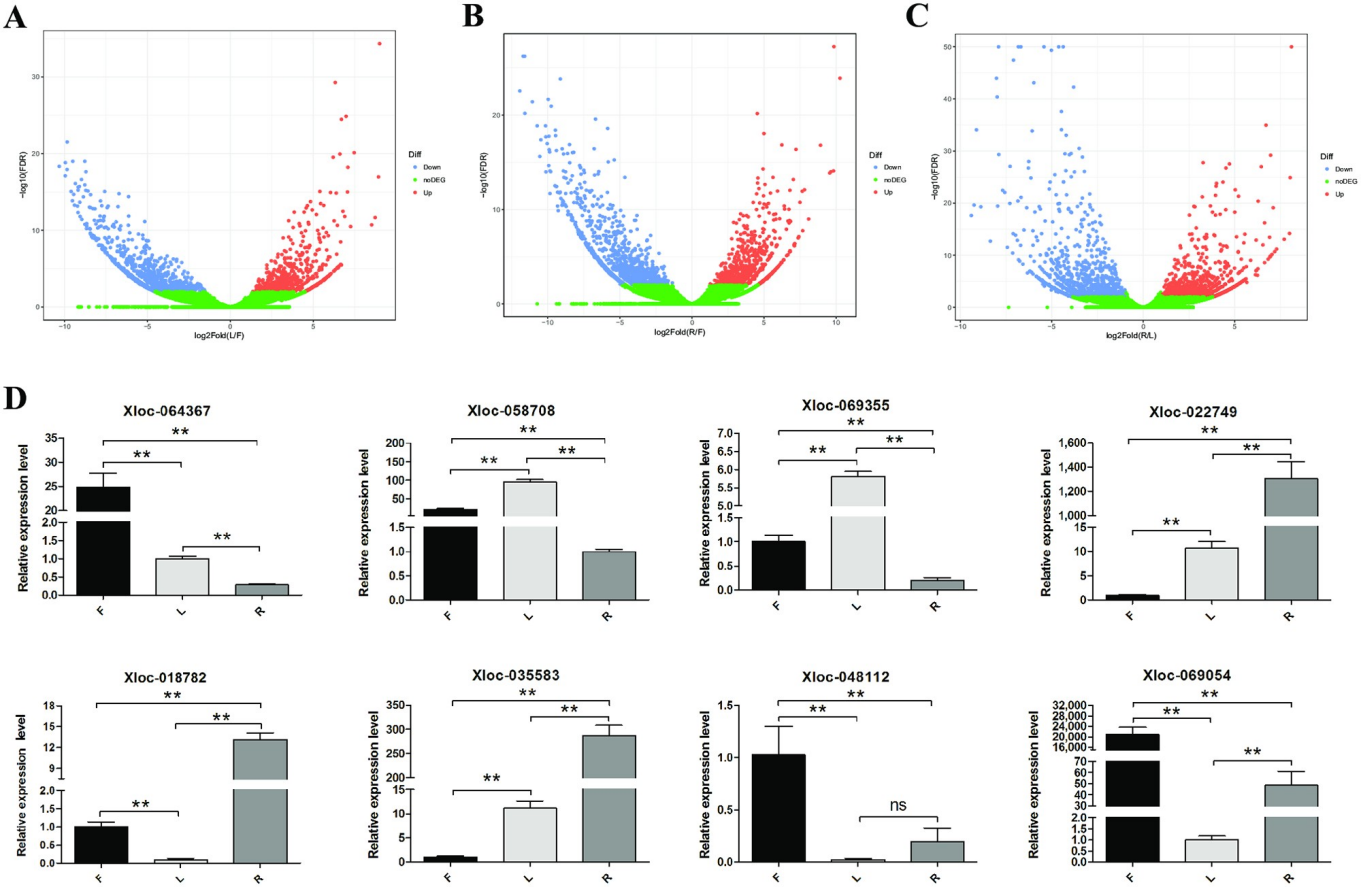
Differential expression volcano analysis and identification of lncRNAs in tomato leaves (L), flowers (F), and roots (R). **(A), (B), and (C)** show the volcano plot of the DE lncRNA genes (DEGs) in the tomato leaves (L), flowers (F), and roots (R). **(A)** is the DEGs_Volcano of F-vs-L, **(B)** is the DEGs_Volcano of F-vs-R, and **(C)** is the DEGs_Volcano of L-vs-R. Red, green, and blue represents up-regulated, down-regulated, and non-DEGs. **(D)** Eight of the 65 most specifically expressed candidate lncRNAs in tomato flowers, leaves, and roots were randomly selected for the qRT-PCR. Actin expression was used as the internal reference. The y-axis shows relative expression analyzed by qRT-PCR. The columns and error bars indicate means and standard deviations of relative expression levels (n = 3), respectively. The error bars are based on three biological replicates, each measured in triplicate. Significant difference (p < 0.05) between the leaf and root was also evaluated. ** represents p < 0.01 and ns represents p > 0.05.

**Fig 4:**
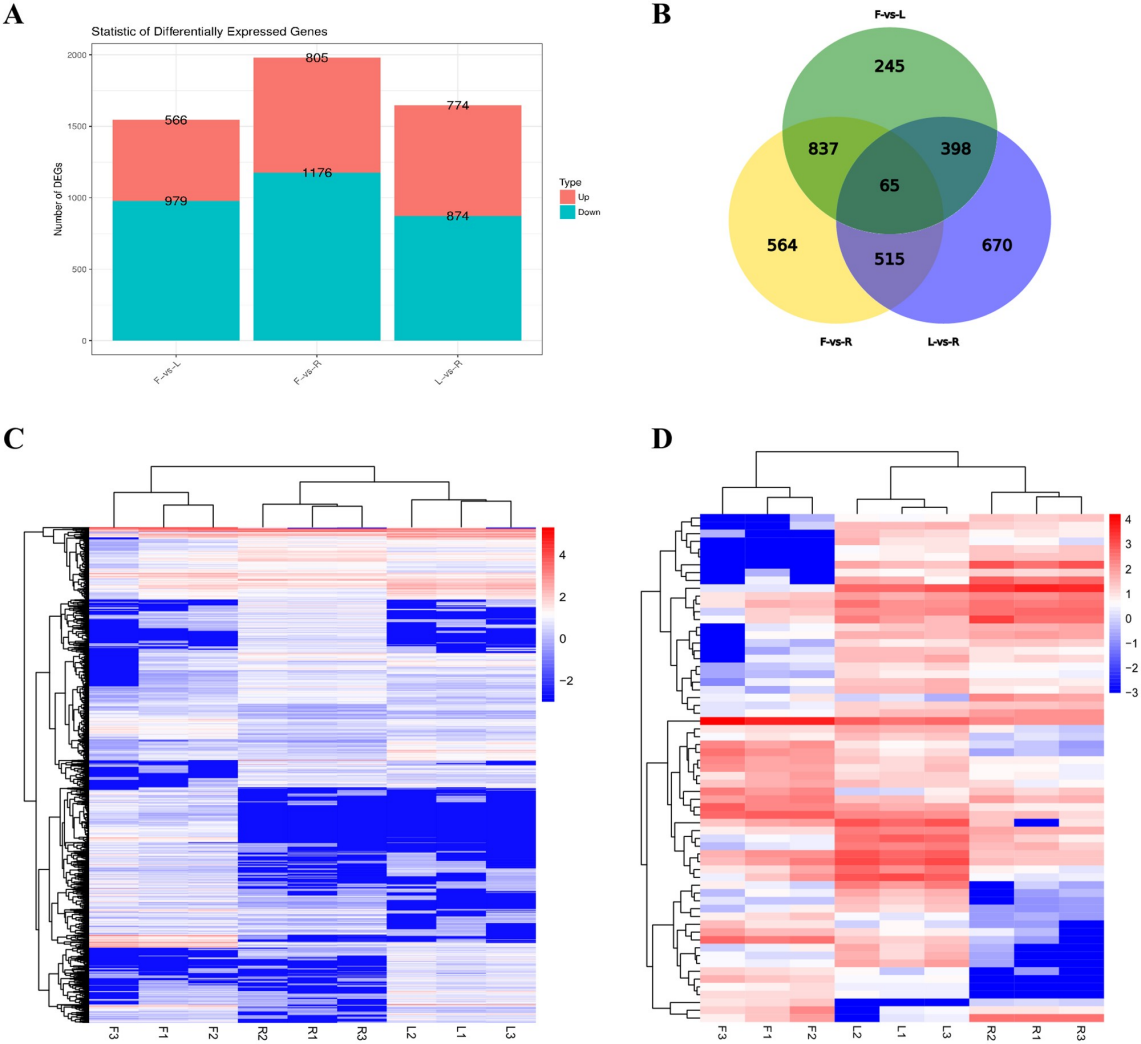
Differential expression patterns of lncRNAs in the tissues of tomato plants. **(A)** Histogram showing DE lncRNAs among tissues of tomato. The number of up-regulated (red) and down-regulated (sky-blue) lncRNAs **is** displayed at the top of each bar. **(B)** Venn diagram showing the number of DE lncRNAs in each tissue of tomato. Hierarchical clustering shows the expression pattern of all the lncRNAs **(C) and DE** lncRNAs among tissues **(D)** were identified in tomato. **The** vertical columns represent different tissues of tomato. **The h**orizontal rows represent **the** lncRNAs. The up-regulated and down-regulated lncRNAs are **represented in** red and blue, respectively.

The expression of tomato lncRNAs in different tissues is shown in the volcano plot (Fig 3A, 3B and 3C). A total of 3294 lncRNAs DE in tomato plants, were obtained; 1545 between flowers and leaves, 1989 between flowers and roots, and 1648 between leaves and roots. Fig 4A shows the number of up-regulated or down-regulated DE lncRNAs. The hierarchical clustering results of 3294 DE lncRNAs are shown in Fig 4C. Sixty-five novel lncRNAs were obtained by the intersection analysis of DE lncRNAs (Fig 4B). Hierarchical clustering showed that the 65 novel lncRNAs were significantly DE in the flowers, leaves, and roots (Fig 4D). Verification of the lncRNAs randomly selected from the 65 DE lncRNAs by qRT-PCR and expression analysis (Fig 3D) consistent with the results of sequencing. These lncRNAs showed significant differences in the flowers, leaves, and roots. For example, the expression of the lncRNAs xloc-064367, xloc-048112, and xloc-069054 in the flowers was > 3 times higher than that in the leaves and roots, whereas the lncRNAs xloc-018782, xloc-022749, and xloc-035583 higher expression in the roots. xloc-058708 and xloc-069355 exhibited higher expression in the leaves.

### Prediction of cis-acting target genes and their function annotation in differential expression of lncRNAs

**Fig 5:**
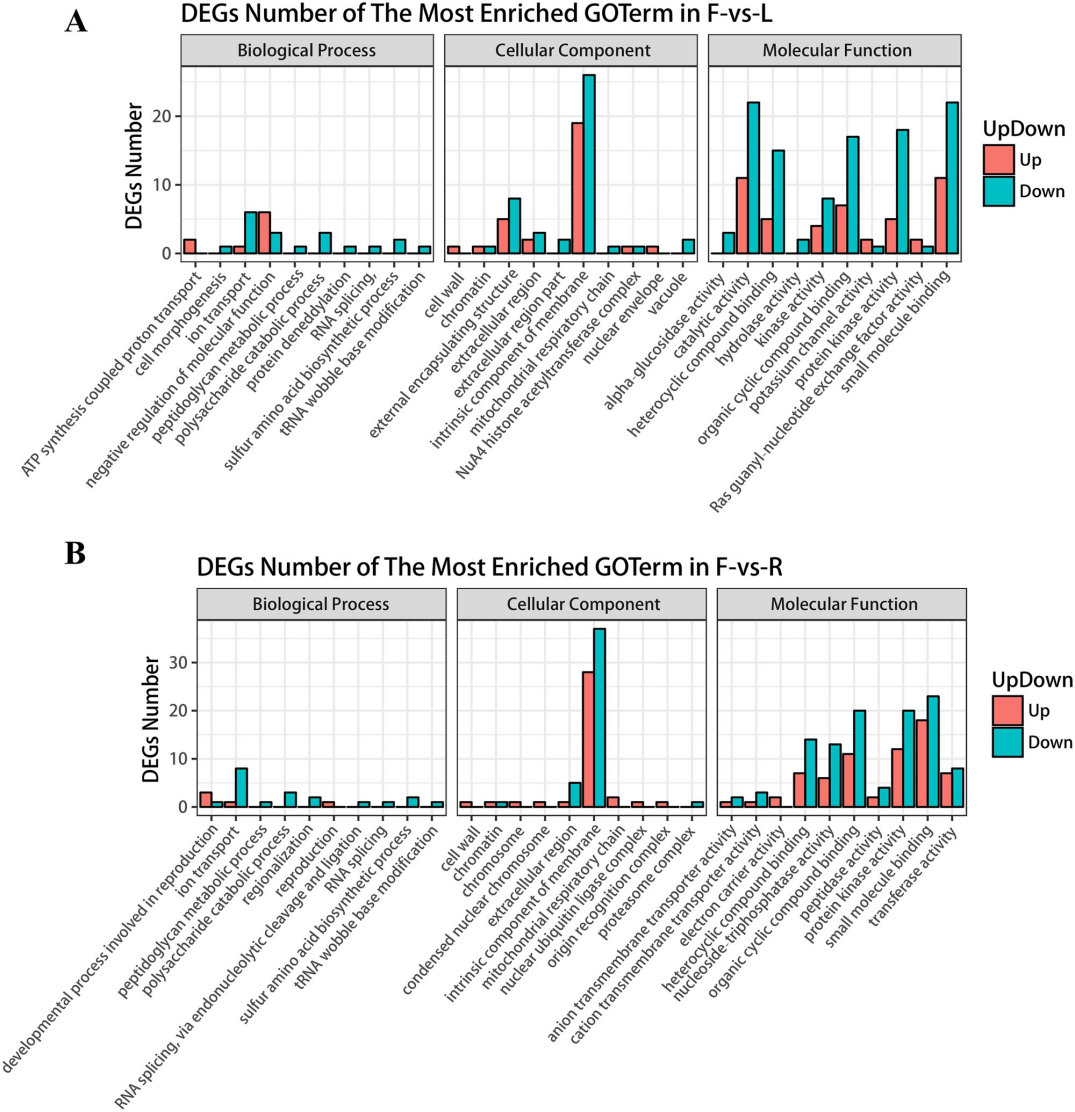
GO Enrichment analysis of DE lncRNA cis target genes in tomato F-vs-L and F-vs-R. **(A)** Gene ontology (GO) enrichment analysis of DE lncRNA cis-target genes in tomato F-vs-L. **(B)** GO enrichment analysis of DE lncRNA cis-target genes in tomato F-vs-R. The y-axis represents the number of DE lncRNA cis-target genes and the x-axis represents the GO functional groups. Red indicates up-regulation of the cis-target gene of lncRNAs and blue indicates down-regulation of the cis-target gene of lncRNAs.

As lncRNAs can regulate the expression of overlapping or nearby genes, we predicted cis-acting target genes of DE lncRNAs (S6 Table), and performed the GO and KEGG enrichment analyses of the cis-acting target genes. The GO analysis (Fig 5) is based on biological process, molecular function, and cellular compartment. Interestingly, unlike the enrichment of DE lncRNA cis-acting target genes in L-vs-R (leaves vs. roots, S1 Fig), the enrichment of DE lncRNA cis-acting target genes in tomato F-vs-L (flowers and leaves, Fig 5A) and F-vs-R (flowers and roots, Fig 5B) was similar. The GO term enriched in the biological process category was mainly derived from the down-regulated lncRNAs, and the enriched GO terms included ion transport, polysaccharide catabolic process, and sulfur amino acid biosynthetic process. The enrichment results of lncRNA in F-vs-L and F-vs-R were similar to the molecular function classification. The cis-acting target genes of the up- or down-regulated lncRNAs were significantly enriched in the intrinsic component of membrane, suggesting that lncRNAs might play a key role in regulating membrane formation in tomato flower tissue. Similar to the enrichment results of molecular functions, we also found a similarity in the enrichment results of the cell components. For instance, in the molecular function category, the enrichment results of the DE lncRNA cis-acting target genes showed that the binding terms (GO:0005488) were the most enriched (such as heterocyclic compound binding, organic cyclic compound binding, and small molecule binding). The above results indicate that lncRNAs might have more important functions in flower tissues than in the leaves and roots during tomato flowering. The lncRNAs might adapt to the rapid growth of flower tissue by regulating the formation of membranes in flower tissues, binding to various molecules, and influencing metabolism.

**Fig 6:**
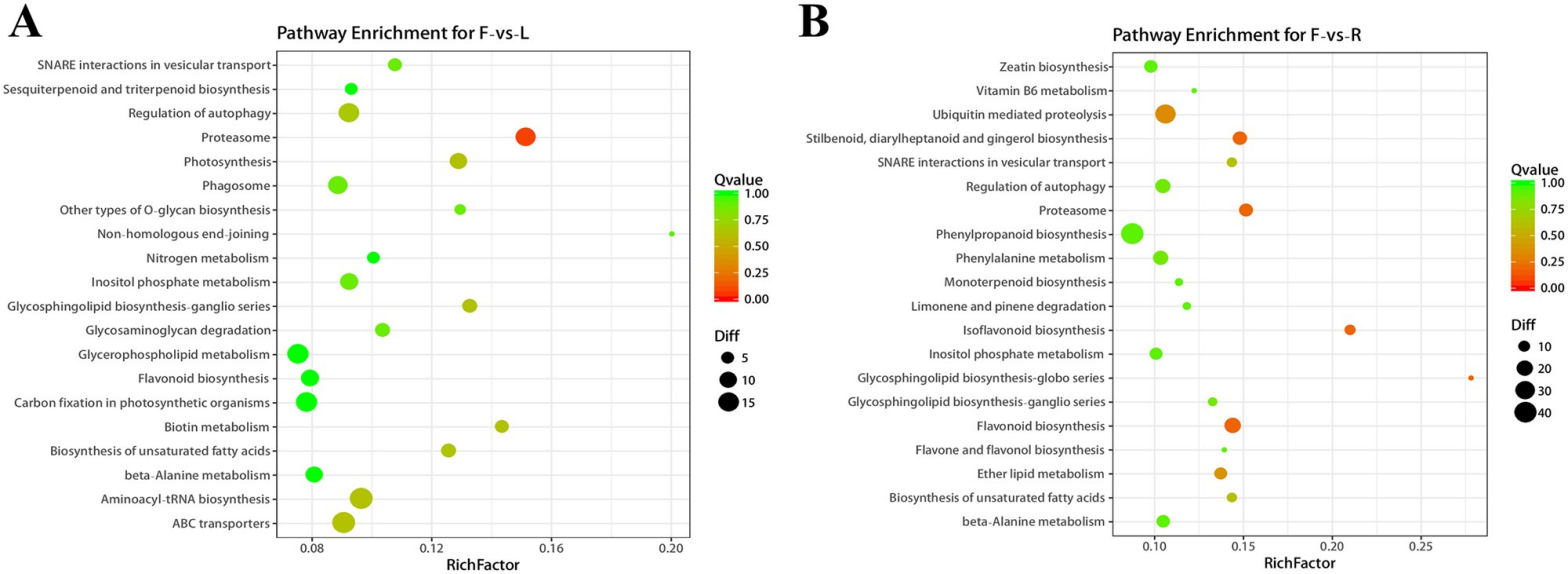
Enrichment of the KEGG pathway of DE lncRNA cis target genes in tomato F-vs-L and F-vs-R. **(A)** The top 20 pathways associated with the cis target mRNAs of DE lncRNAs in tomato F-vs-L are listed. **(B)** The top 20 pathways associated with the cis target mRNAs of DE lncRNAs in tomato F-vs-R are listed. The enrichment Q value or false discovery rate corrected the p value for multiple comparisons. The P values were calculated using Fisher’s exact test. The item/pathway on the vertical axis was drawn according to the first letter of the pathway in descending order. The horizontal axis represents the enrichment factor. The top 20 enriched pathways were selected according to the enrichment factor value. The different colors from green to red represent the Q value (false discovery rate value). The different sizes of the round shapes represent the number of genes in a pathway.

The results of the KEGG enrichment analysis (Fig 6) showed that DE lncRNA cis-acting target genes of different tissues were mainly enriched in the basic synthetic and metabolic pathways, such as the biosynthesis of unsaturated fatty acids, flavonoid biosynthesis, and glycosphingolipid biosynthesis in the synthetic pathway; and glycerophospholipid metabolism, inositol phosphate metabolism, and beta-alanine metabolism in the metabolic pathways, which are essential for the normal growth and development of tomato during flowering. Simultaneously, we observed that DE lncRNA cis target genes were highly enrichment in the phagosome pathway and regulation of autophagy. This indicates that lncRNAs might affect the apoptosis of tomato flower tissues by regulating the ubiquitin–proteasome pathway (UPP) and regulate flower decline and fruit formation in tomato. These data suggest that lncRNAs might play an important role in the growth, development, and apoptosis of flowering period tomato plant.

### Analysis of ceRNA network

**Fig 7:**
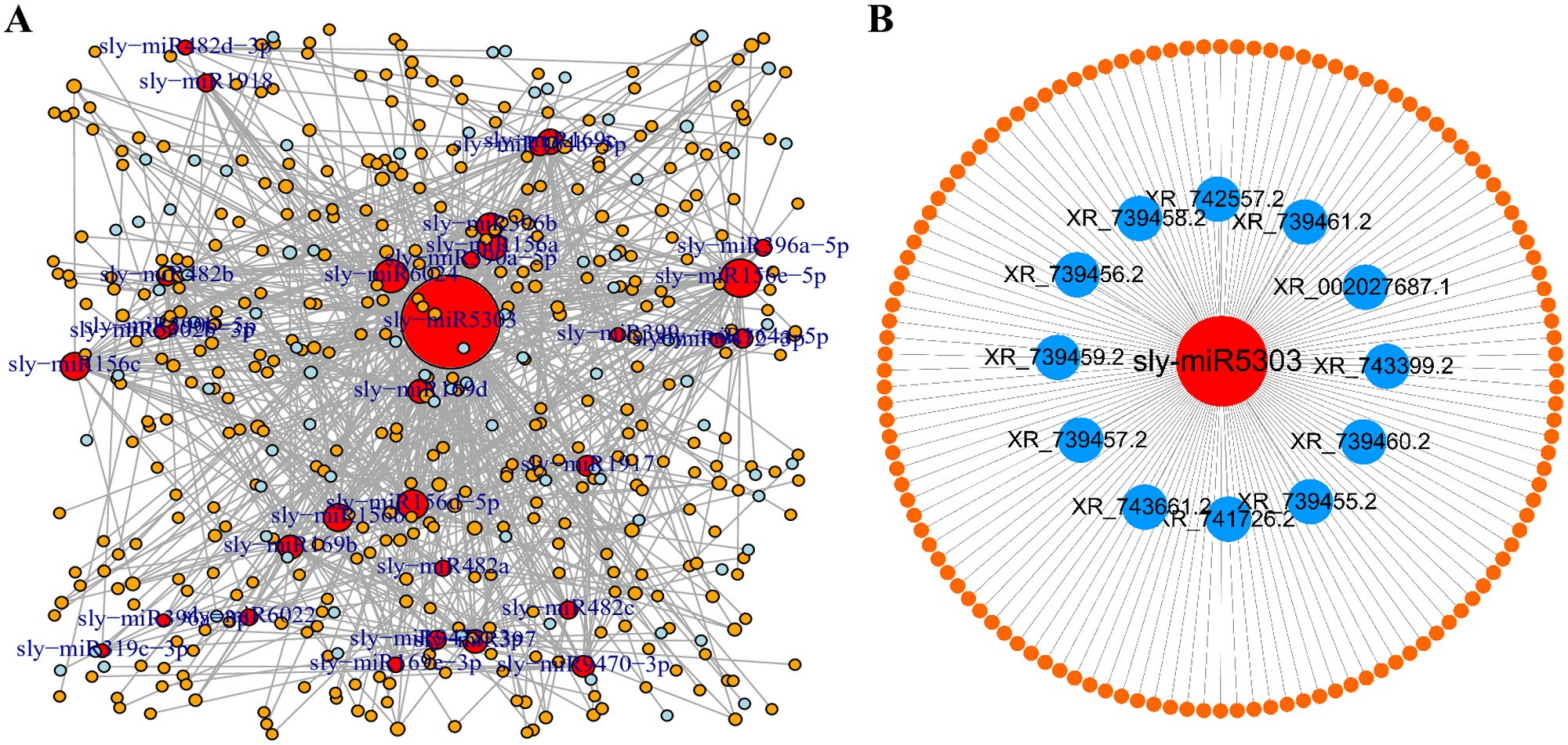
lncRNA-miRNA-mRNA interaction network in tomato. The sky-blue, red, and orange nodes denote lncRNAs, miRNAs, and genes, respectively. Each gray edge denotes a target relationship between a miRNA and a gene or lncRNA, hidden in the ceRNA network. **(A)** The panorama network consists of 78 lncRNAs, 32 miRNAs, and 397 target mRNAs in tomato. **(B)** A subnetwork comprising sly-miR5303, 12 lncRNAs, and their target mRNAs in tomato.

As lncRNAs can combine with the miRNAs to inhibit its interaction with mRNA competitively, they play a regulatory role in the growth and development of plants [38–40]. In this study, we predicted the miRNA targets on lncRNA and mRNA, and the correlation network of lncRNA-miRNA-mRNA was constructed (Fig 7A). The network showed the interaction between 32, 78, and 397 kinds of miRNAs, lncRNAs, and mRNAs, respectively (S7 Table). For example, Sly-miR164b-5p can be combined with lncRNA XR_002027860.1 and mRNA XM_010327149.2, whereas lncRNA XR_740288.2 and XR_002027182.1 can affect the expression of XM_010318452.2, XM_010318450.2, XM_004232829.3, and XM_010318451.2 through sly-miR399. The ceRNA regulation mode that combines miRNA was not aimed at a single lncRNA or mRNA. Fig 6A shows that a miRNA could be associated with one or many mRNAs and vice versa. For example, sly-miR5303 can interact with 12 lncRNAs and 126 mRNAs to participate in a variety of metabolic pathways during the development of tomato (Fig 7B). However, we found that the regulatory model of ceRNA has synergism and that miRNAs of the same family are more likely to play the same type of ceRNA function. For example, sly-miR156a, sly-miR156b, sly-miR156c, sly-miR156d, and sly-miR156e can interact with XM_004238983.3, XM_010322704.2, and XM_004243319.2, and lncRNA XR_002026094.1 can form ceRNA with sly-miR156a and sly-miR156b simultaneously. These findings indicate that the expression profiles of miRNA, mRNA, and lncRNA are significantly correlated.

## Discussion

Previous studies have shown that the lncRNA can act as scaffolds or guides for the regulation of gene expression via epigenetic modifications or other post-transcriptional regulation mechanisms [4, 57–59]. The lncRNAs play a key role in several biological processes of plants [60] and some lncRNAs related to tomato fruit ripening have been identified [61]. Studies on lncRNA associated with flowering are of great significance for tomato growth and physiological metabolism. In this study, 10,919 lncRNAs containing 248 novel lncRNAs were identified, and 65 lncRNAs were significantly DE in the flowers, leaves, and roots. A large number of DE lncRNAs that were associated with metabolic processes and enzyme activities was identified. This suggests that lncRNA might play an important role during the flowering stage in tomatoes.

While analyzing the characteristics of tomato lncRNAs, we found that the distribution of exons of mRNA and lncRNA in our study was different from that reported by Zhu et al. [61]. Their study in ripe tomato fruit showed that most genes encoding tomato lncRNA contain only one or two exons, whereas the number of exons in genes encoding proteins ranged from 1 to 10. In our study, although the exon distribution of mRNA was similar to that previously reported, the distribution of lncRNAs was quite different. The number of lncRNA transcripts with a single exon was fewer, whereas more than 90% of lncRNAs contained more than two exons (Fig 2C). This showed that tomato might produce different lncRNAs to regulate the growth and development of plant at different developmental stages. After categorizing the lncRNAs, we found that although the number of lncRNAs decreased with the increase in tomato lncRNA length, the same-strand type was the most common of the six types and clearly occupied a higher proportion in the short lncRNA (< 1500 bp). This suggests that tomato might be more likely to form shorter subset strand type lncRNAs to regulate the development of tomato during the flowering stage.

Similar to the findings of Deng et al. [62], flowering period tomato lncRNAs have high tissue specificity. We found 3294 lncRNAs with significant differential expression in tomato leaves, flowers, and roots (Fig 3A). In the GO enrichment analysis of DE lncRNA cis-target genes, we found that the GO enrichment results of DE lncRNA cis target genes in F-vs-L and F-vs-R were similar. This phenomenon might be due to the fact that the flower tissue is not the tissue inherent throughout the life cycle of the tomato plant, but occurs at a specific growth stage before the tomato fruit is produced. Once the fruit is produced, the flower will quickly wilt; therefore, it requires specific lncRNAs to regulate this process. For example, the GO-enriched terms included binding, intrinsic component of membrane, and polysaccharide catabolic process, indicating the important role of lncRNAs in the formation and metabolism of flower tissues. Moreover, in the KEGG pathway enrichment, we found that the DE lncRNA cis target genes have a higher enrichment degree in the phagosome pathway and regulation of autophagy. This further indicates that lncRNAs can also regulate the apoptosis process of tomato tissues. In summary, we found that lncRNAs play an irreplaceable role in the process of tomato flower tissue, from generation to apoptosis. This is similar to the findings of Wang et al. [63] in *Catalpa bungei* and Huang et al. [64] in *Brassica rapa* pollen.

Several studies have suggested that lncRNA can act as a ceRNA to inhibit the function of miRNAs and compete with mRNA, pseudogenes, and other miRNA sponges for miRNA [39, 65–66]. In the present study, the lncRNA-miRNA-mRNA-related network was constructed based on the ceRNA action principle. We found that 78 lncRNAs and 397 miRNAs have 32 miRNA binding sites. For example, mRNA XM_019212257.1 could act as an eTM of sly-miR156b, mRNA XM_010322716.2 could act as an eTM of sly-miR156c, whereas lncRNA XR_002026094.1 could act as a ceRNA of sly-miR156b and sly-miR156c, simultaneously. This suggests that lncRNA may be involved in the development of flowering tomato plants by forming ceRNA. Further studies on how tomato lncRNAs are used as ceRNAs to regulate the development of flowering tomato are needed to verify the underlying mechanisms.

In this study, we analyzed the expression characteristics of lncRNA during the tomato flowering stage and identified a series of lncRNAs with significant differences in different tomato plant tissues. By analyzing the GO and KEGG data of differential lncRNA cis-target genes, we found that the lncRNAs might play an important role at the tomato flowering stage. Simultaneously, we also found that lncRNA might affect the development of flowering tomato by forming ceRNA. The results substantially improve our understanding of the lncRNAs during the development of flowering tomato plants. The study lays a foundation for further research on the lncRNA function during the tomato flowering stage.

## Supporting information

S1 Fig Classification of lncRNAs. **(A)** lncRNA located in a gene (Genic) can be divided into containing, convergent, and nested types; **(B)** intergenic lncRNAs can be divided into divergent, same strand, and overlapping types. The solid line frame displays lncRNA partner genes and the dotted line frame illustrates the lncRNAs.
(PDF)

S2 Fig Enrichment of the GO and KEGG pathway of DE lncRNA cis target genes in tomato L-vs-R and F-vs-R. **(A)** GO enrichment analysis of DE lncRNA cis-target genes in tomato L-vs-R. **(B)** The top 20 pathways associated with the cis target mRNAs of DE lncRNAs in tomato F-vs-L are listed.
(PDF)

S1 Table qRT-PCR primers of lncRNA. (XLSX)

S2 Table RNA-seq data for nine samples. (XLSX)

S3 Table Newly predicted lncRNA. (XLSX)

S4 Table lncRNA of three tomato tissues. (XLSX)

S5 Table Novel lncRNA of three tomato tissues. (XLSX)

S6 Table lncRNA cis target gene. (XLSX)

S7 Table Network relationship of lncRNA-miRNA-mRNA. (XLSX)
