# Supplementary figures and images for: LncRNA expression profile and ceRNA analysis in tomato during flowering
Source: PLoS One. 2019 Jan 17;14(1):e0210650. doi: 10.1371/journal.pone.0210650 (PMC6336255; doi:10.1371/journal.pone.0210650)

**A**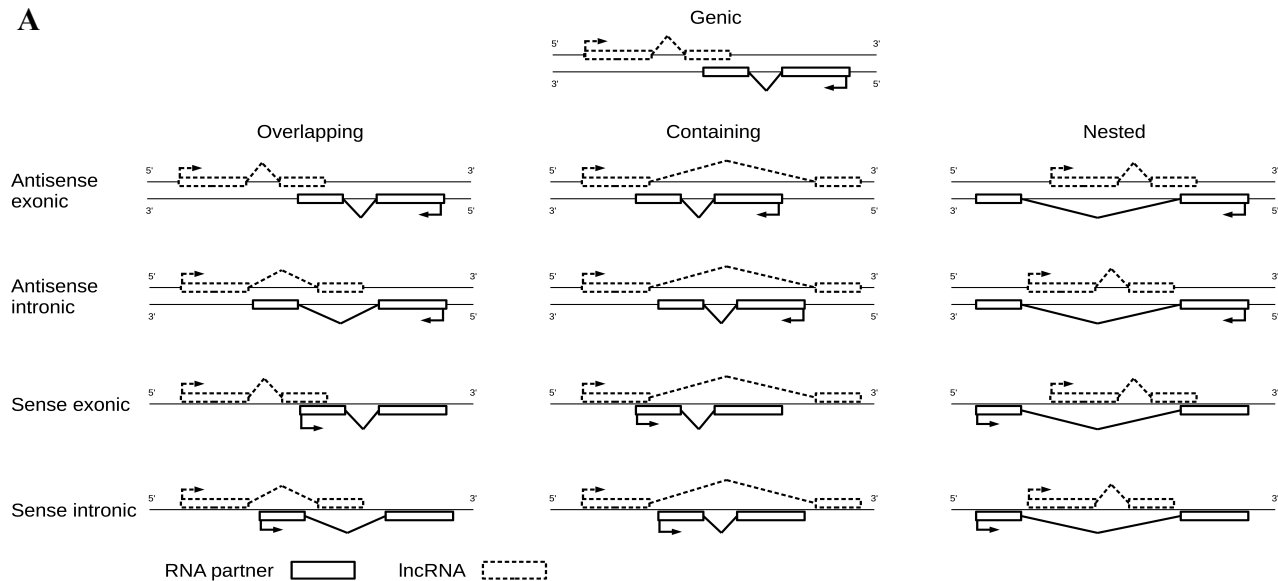**B**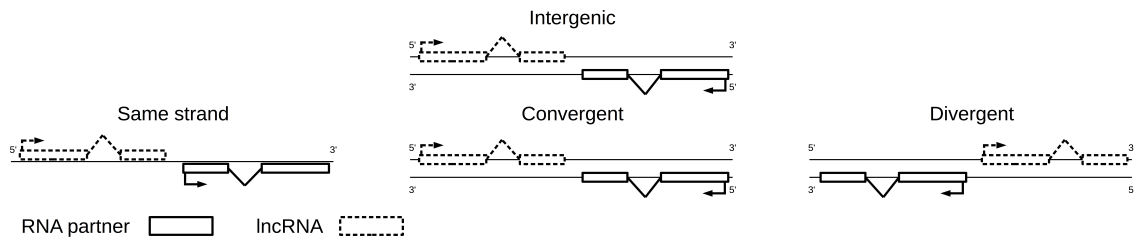

Supplement: S1 Fig — (A) lncRNA located in a gene (Genic) can be divided into containing, convergent, and nested types; (B) intergenic lncRNAs can be divided into divergent, same strand, and overlapping types. The solid line frame displays lncRNA partner genes and the dotted line frame illustrates the lncRNAs. (PDF) [file pone.0210650.s001.pdf]

A

## DEGs Number of The Most Enriched GO Term in L-vs-R

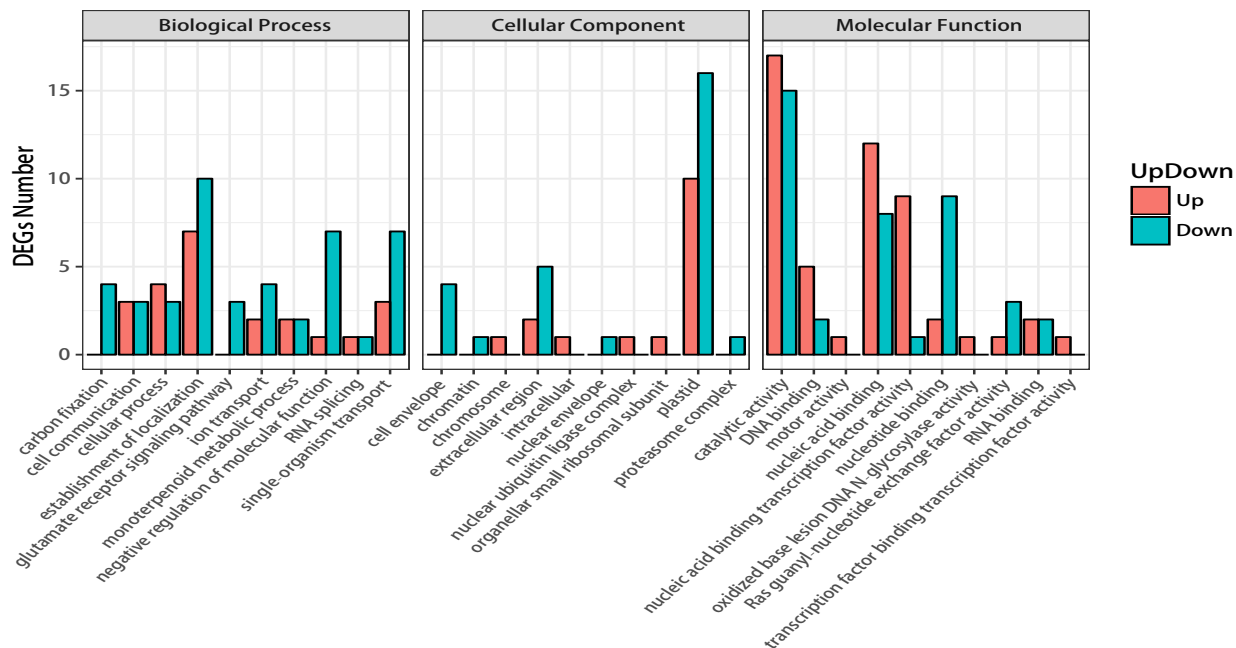

B

## Pathway Enrichment for L-vs-R

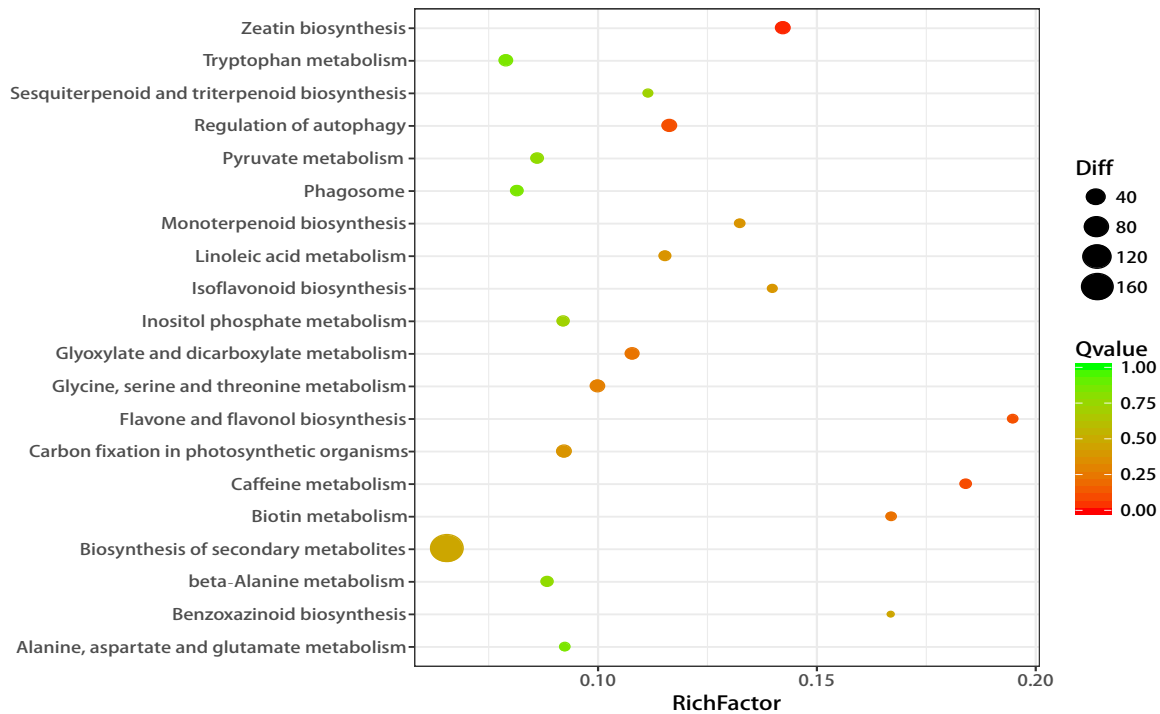

Supplement: S2 Fig — (A) GO enrichment analysis of DE lncRNA cis-target genes in tomato L-vs-R. (B) The top 20 pathways associated with the cis target mRNAs of DE lncRNAs in tomato F-vs-L are listed. (PDF) [file pone.0210650.s002.pdf]
